# Communication Technology Preferences of Hospitalized and Institutionalized Frail Older Adults During COVID-19 Confinement: Cross-Sectional Survey Study

**DOI:** 10.2196/21845

**Published:** 2020-09-18

**Authors:** Guillaume Sacco, Sébastien Lléonart, Romain Simon, Frédéric Noublanche, Cédric Annweiler, TOVID Study Group

**Affiliations:** 1 Department of Geriatric Medicine and Memory Clinic University Hospital of Angers Angers France; 2 Laboratoire de Psychologie des Pays de la Loire (LPPL - EA 4638) SFR Confluences Université Nantes-Angers-Le Mans Nantes France; 3 ALLEGRO Living Lab Research Center on Autonomy and Longevity University Hospital of Angers Angers France; 4 Pôle médico-social Saint Nicolas Univeristy Hospital of Angers Angers France; 5 Robarts Research Institute, Department of Medical Biophysics Schulich School of Medicine and Dentistry University of Western Ontario London, ON Canada; 6 School of Medicine Health Faculty University of Angers Angers France

**Keywords:** video communication, telephone, older adults, nursing home, hospital, confinement, elderly, COVID-19, communication, technology, social isolation, loneliness

## Abstract

**Background:**

Technological communication methods such as telephone calls and video calls can help prevent social isolation and loneliness in frail older adults during confinement.

**Objective:**

Our objectives were to determine which virtual communication method (ie, telephone call or video call) was preferred by confined older hospital patients and nursing home residents and the variables influencing this preference.

**Methods:**

The TOVID (Telephony Or Videophony for Isolated elDerly) study was a cross-sectional study that was designed to examine the preference between telephone calls and video calls among frail older adults who were either hospitalized in a geriatric acute care unit or institutionalized in a long-term care and nursing home during the COVID-19 confinement period.

**Results:**

A total of 132 older people were surveyed between March 25 and May 11, 2020 (mean age 88.2 years, SD 6.2); 79 (59.8%) were women. Patients hospitalized in the geriatric acute care unit were more able to establish communication independently than residents institutionalized in the long-term care and nursing home (*P*=.03) and were more satisfied with their communication experiences (*P*=.02). Overall, older people tended to favor telephone calls (73/132, 55.3%) over video calls (59/132, 44.7%); however, their satisfaction degree was similar regardless of the chosen method (*P*=.1), with no effect of age (*P*=.97) or gender (*P*=.2). In the geriatric acute care unit, the satisfaction degrees were similar for telephone calls (40/41, 98%) and video calls (33/38, 87%) in older patients (*P*=.10). Conversely, in the long-term care and nursing home, residents were more satisfied with the use of video calls to communicate with their relatives (14/15, 93%) versus the use of telephone calls (6/12, 50%; *P*=.02).

**Conclusions:**

Older people confined to health care settings were able to complete telephone calls more independently than video calls, and they tended to use telephone calls more often than video calls. The satisfaction degrees were similar with both modalities and even greater with video calls among long-term care and nursing home residents when they were given assistance to establish communication.

**Trial Registration:**

ClinicalTrials.gov NCT04333849: https://www.clinicaltrials.gov/ct2/show/NCT04333849.

## Introduction

All serious epidemics, such as the COVID-19 pandemic, prompt social organizations to deeply rethink the services they offer, especially toward frail older adults. For instance, the visitation restrictions in geriatric care units and nursing homes, although essential to the control of the pandemic, have also become a major source of social isolation and loneliness for vulnerable populations [1-3]. The only remaining social link for patients and residents in these facilities during confinement has been virtual communication using a range of communication devices, notably telephone calls and video calls.

The use of video calls is currently increasing because of the innovative nature of video, which allows people to speak to and hear others, view their expressions, and establish richer relationships than may be possible with a simple telephone call [4]. The research question in this paper is whether video calling meets the real demands and expectations of older adults or whether they prefer more traditional communication methods, such as the telephone**,** to contact their relatives. Moreover, evidence from previous literature about the efficacy of electronic interventions to avoid social isolation among older adults is weak and inconsistent [5-7].

Like many other practitioners, from the start of the COVID-19 public health crisis and the first visit restrictions for hospitalized and institutionalized patients, we proposed to organize daily communications between the patients and their relatives to avoid excessive isolation [8,9]. Both telephone calls and video calls were offered to the patients. The main objective of this study was to determine which virtual communication mode was preferred by older patients confined in hospital or institutionalized in a nursing home. Our secondary aims were to identify the proportions of older patients and residents who could independently communicate with virtual support; to measure and compare the degrees of satisfaction following telephone calls and video calls; and to analyze the effects of age and context (hospitalization versus nursing home) on the communication mode choice of older adults.

## Methods

### Design and Settings

The cross-sectional TOVID (Telephony Or Videophony for Isolated elDerly) study was conducted in the geriatric acute care unit and in the long-term care unit and nursing home of the University Hospital of Angers, France, between March 25 and May 11, 2020, during the national confinement period in France. No outside visits to these hospital units were authorized during this period. The study was conducted in accordance with the ethical standards set forth in the Helsinki Declaration (1983) and was approved by the local ethics committee (number 2020/29). The study protocol was declared to the National Commission for Information Technology and Civil Liberties (CNIL) under the number ar20-0030v0 (ClinicalTrials.gov identifier: NCT04333849).

### Participants

All older adults consecutively hospitalized in the geriatric acute care unit and in the long-term care and nursing home were considered for inclusion in the study. Patients who refused to participate or who were unable to communicate with their relatives for medical reasons were not included in the study.

### Data Collection

Health professionals accustomed to using communication devices visited all eligible patients at least once per day to offer to help them organize their communications with their relatives. All patients who expressed interest were offered either a telephone call or a video call, and they were clearly informed that they could receive assistance to establish communication if necessary. All cognitively intact patients who objected to any help were considered to be independently capable of establishing communication (ie, declarative measure). The details of the communication (application and equipment, schedule, and duration) were discussed with the relatives prior to the communication to ensure that the call proceeded smoothly and easily.

Data regarding the participants’ age, gender, hospital unit (ie, geriatric acute care unit or long-term care and nursing home), independent ability to establish communication, preferred virtual communication mode, and degree of satisfaction toward the mode of communication were collected. The preferred virtual communication mode was identified using a standardized question with three options (nothing, telephone call, or video call). The satisfaction degree toward virtual communication was assessed after the communication using a 6-point Likert scale (with 1=not satisfied at all to 6=totally satisfied), and the proportion of people satisfied with the communication (defined as a score ≥5/6) was analyzed.

### Number of Participants

Because the main objective of the study was descriptive, it was not necessary to calculate the number of subjects. However, to match a normal distribution of the data and to use parametric statistical tests, at least 30 participants were required in each group (ie, n=30 in the telephone call group and n=30 in the video call group).

### Statistical Analyses

Categorical variables were described using numbers and percentages and quantitative variables were described using means and standard deviations, as appropriate. Comparisons between older patients in the geriatric acute care unit and the long-term care and nursing home and between patients who chose telephone calls and video calls were performed using chi-square tests for qualitative variables (or the exact Fisher test where appropriate), and the Student *t* test was used for quantitative variables (or the Mann-Whitney U test where appropriate). *P* values <.05 were considered significant. All statistical analyses were performed with SPSS version 20 (IBM Corporation).

### Availability of Data and Materials

Patient level data are freely available from the co-corresponding author at Cedric.Annweiler@chu-angers.fr after notification and authorization of the competent authorities. There is no personal identification risk with these anonymized raw data.

## Results

We invited 163 older adults to take part in the study; 132 (80.1%) agreed to participate and were included in the study. The age range of the 132 participants was 66 to 103 years (mean age 88.2 years, SD 6.2); 78 (59.1%) were women.

As illustrated in Table 1, the participants tended to favor telephone calls (73/132, 55.3%) over video calls (59/132, 44.7%). The satisfaction degrees with the two modalities were similar (46/73, 87%, with telephone calls versus 47/59, 89%, with video calls, *P*=.10). There was no effect of age (**P*=.97*) or gender (*P*=.16) on the choice of virtual communication mode.

Patients hospitalized in the geriatric acute care unit were more frequently able to independently establish communication (24/105, 22.8%) than residents institutionalized in the long-term care and nursing home (1/27, 3.8%, *P*=.03) and they were more able to independently establish communication by telephone call (22/73, 30.1%) than by video call (3/59, 5.1%; *P*<.001). Moreover, patients hospitalized in the geriatric acute care unit were more often satisfied with the communication (73/79, 92%) than residents of the long-term care and nursing home (20/27, 74%; *P*=.02).

**Table 1:** Characteristics of the study participants (N=132).

Characteristic		Whole cohort	Preferred virtual communication mode			Location of confinement		
		(N=132)	Telephone call (n=73)	Video call (n=59)	*P* value^a^	Geriatric acute care unit (n=105)	Long-term care and nursing home (n=27)	*P* value^a^
**Sociodemographic data**								
	Age (years), mean (SD)	88.2 (6.2)	88.2 (5.7)	88.2 (6.7)	.97	88.8 (5.4)	85.8 (8.1)	.08
	Female gender, n (%)	78 (59.1)	39 (53.4)	39 (66.1)	.16	57 (54.3)	21 (77.8)	.03
**Communication with relatives, n (%)**								
	Capability of independently establishing communication^b^	25 (19.1)	22 (30.1)	3 (5.2)	<.001	24 (22.8)	1 (3.8)	.03
	Choice of telephone call	73 (55.3)	N/A^c^	N/A	N/A	61 (58.1)	12 (44.4)	.28
	High degree of satisfaction (Likert scale score ≥5/6)^d^	93 (87.7)	46 (86.8)	47 (88.7)	>.99	73 (92.4)	20 (74.1)	.02

^a^Comparisons based on chi-square test or exact Fisher test for qualitative variables and Student *t* test or Mann-Whitney U test for quantitative variables, as appropriate.

^b^Data missing for 1 participant in the long-term care and nursing home.

^c^N/A: not applicable.

^d^Data missing for 26 participants in the geriatric acute care unit.

In the subgroup analyses (Table 2), older patients chose telephone calls and video calls at similar frequencies when they were hospitalized in the geriatric acute care unit (61/106, 57.5% and 44/106, 41.5%, respectively) or institutionalized in the long-term care and nursing home (12/27, 44.4%, and 12/27, 55.6%, respectively). In the geriatric acute care unit, the satisfaction degrees were similar for telephone calls (40/41, 98%) and video calls (33/38, 87%) in older patients (*P*=.10). Conversely, in the long-term care and nursing home, residents were more often satisfied with the use of video calls to communicate with their relatives (14/15, 93%, versus 6/12, 50%, *P*=.02) (Table 2).

**Table 2:** Subgroup analyses according to confinement place (N=132).

Characteristic		Patients in the geriatric acute care unit				Residents in the long-term care and nursing home		
		Choice of telephone call (n=61)	Choice of video call (n=44)	*P* value^a^	Choice of telephone call (n=12)		Choice of video call (n=15)	*P* value^a^
**Sociodemographic data**								
	Age (years), mean (SD)	88.5 (5.6)	89.3 (5.2)	.49	86.8 (6.1)		85.0 (9.6)	.59
	Female gender, n (%)	31 (51)	26 (59)	.43	8 (67)		13 (87)	.36
**Communication with relatives, n (%)**								
	Capability of independently establishing communication^b^	22 (36)	2 (5)	<.001	0 (0)		1 (7)	>.99
	High degree of satisfaction (≥5/6)^c^	40 (98)	33 (87)	.10	6 (50)		14 (93)	.02

^a^Comparisons based on chi-square test or exact Fisher test for qualitative variables and Student *t* test or Mann-Whitney U test for quantitative variables, as appropriate.

^b^Data missing for 1 participant in the long-term care and nursing home.

^c^ Data missing for 26 participants in the geriatric acute care unit.

## Discussion

### Principal Results

We found that older adults confined to health care settings (ie, a geriatric acute care unit or long-term care and nursing home) were more often independently able to perform telephone calls than video calls, and they tended to use the telephone more often to communicate with their relatives. Their levels of satisfaction were similar with both communication supports, and satisfaction was even greater with video calls among residents of the long-term care and nursing home when they received assistance to establish communication.

### Limitations

Our study has some limitations. First, the study was monocentric, which limits the representativeness of the study population even if we were able to include a relatively high number of participants. Second, the results should be interpreted with caution because some confounding factors such as cognition and mood were not assessed. Larger, and if possible prospective, studies should be conducted on different population groups to better understand the need for video calls and their effects on loneliness, social isolation, and quality of life in older adults.

### Comparison With Prior Work

The global confinement during the COVID-19 pandemic has highlighted the importance of preventing social isolation and loneliness in older adults [1,2], as social disconnection is associated with increased anxiety and depression [10] and loneliness is associated with increased risks of health disorders, including major neurocognitive disorders [11]. Access to social technologies has been widely proposed to reduce social isolation during the pandemic [8,12], although uncertainty remains about efficacy of video call interventions to reduce loneliness in older adults [13]. Despite this, studies focusing on these technological interventions among frail older adults remain rare [14], and to our knowledge, no study has evaluated the acceptance and preferences of older adults regarding these interventions. However, there is some evidence that a weak digital culture and eventual impairments may complicate the use of virtual communication modes by older adults [15].

Technological acceptance is a balance between perceived usefulness and perceived simplicity of use [16], to which is added the quality of the output (ie, the way in which the system performs the expected task [17]). A model of technology acceptance dedicated to older adults brings together 10 factors influencing the acceptance of the technology: the value (utility), usability (perceived simplicity), affordability, accessibility, technical support, social support, emotion (output quality), independence, experience, and confidence [15]. In our study, pre-experience (ie, perceived utility and perceived simplicity of use, reflected by the choice of communication mode) and post-experience perceptions (ie, output quality, reflected by the satisfaction degree) were different between geriatric acute care unit patients and long-term care and nursing home residents. In our study, the preference for telephone calls (with 55% of participants making this choice) is likely due to the participants’ pre-experience of the perceived simplicity of this device compared to video calls, which are more often misunderstood and less often used by people in this older generation. In addition, the postexperience perception differed between geriatric acute care unit patients and long-term care and nursing home residents, with a higher degree of satisfaction in the geriatric acute care unit than in the long-term care and nursing home (73/29, 92%, vs 20/27, 74%; *P*=.02). One possible explanation is that the proportion of older adults who were unable to establish communication by themselves was higher in the long-term care and nursing home (25/26, 96%) than in the geriatric acute care unit (81/105, 77.1%; *P*=.03). It therefore appears that the pre-experience was unbalanced by the importance given to the perceived ease of use compared to the perceived usefulness of adding video to the call. On the other hand, when older adults in the long-term care and nursing home chose the video calls, generally with assistance establishing the communication, their satisfaction degree was higher with the video calls, which shows that their post-experience was modified and could possibly modify their future choices and communication habits.

We also found that a greater proportion of older adults needed assistance to use video calls (56/59, 94.8%) than to use telephone calls (51/73, 69.9%, *P*<.001). Previous literature has emphasized the importance of offering accessible communication systems, and sustained efforts should be pursued to simplify communication technologies, particularly video calls [14,18]. It would also be interesting to support or adapt these communication technologies for people who have communication issues. Technology may provide support for this.

### Conclusions

Older adults in a geriatric acute care unit and a long-term care and nursing home were more independently able to make telephone calls than video calls, and they tended to use the telephone more often than video. However, their post-experience satisfaction with video calls was high. This cross-sectional study contributes to understanding the acceptance and the challenges of frail older adults regarding video calls.

## Abbreviations

CNIL: National Commission for Information Technology and Civil Liberties
TOVID: Telephony Or Videophony for Isolated elderly

## References

[1] Armitage R, Nellums LB (2020). COVID-19 and the consequences of isolating the elderly. Lancet Public Health.

[2] Cudjoe TK, Kotwal AA (2020). "Social Distancing" Amid a Crisis in Social Isolation and Loneliness. J Am Geriatr Soc.

[3] Brooks SK, Webster RK, Smith LE, Woodland L, Wessely S, Greenberg N, Rubin GJ (2020). The psychological impact of quarantine and how to reduce it: rapid review of the evidence. Lancet.

[4] Hemberg J, Santamäki Fischer R (2018). A Window Toward the World Older Adults' Experiences of Becoming in Health and Developing as Human Beings Through Interacting With Others Using Real Video Communication. Holist Nurs Pract.

[5] Chipps J, Jarvis MA, Ramlall S (2017). The effectiveness of e-Interventions on reducing social isolation in older persons: A systematic review of systematic reviews. J Telemed Telecare.

[6] Gardiner C, Geldenhuys G, Gott M (2018). Interventions to reduce social isolation and loneliness among older people: an integrative review. Health Soc Care Community.

[7] Poscia A, Stojanovic J, La Milia DI, Duplaga M, Grysztar M, Moscato U, Onder G, Collamati A, Ricciardi W, Magnavita N (2018). Interventions targeting loneliness and social isolation among the older people: An update systematic review. Exp Gerontol.

[8] Eghtesadi M (2020). Breaking Social Isolation Amidst COVID-19: A Viewpoint on Improving Access to Technology in Long-Term Care Facilities. J Am Geriatr Soc.

[9] Ruopp MD (2020). Overcoming the Challenge of Family Separation From Nursing Home Residents During COVID-19. J Am Med Dir Assoc.

[10] Santini ZI, Jose PE, York Cornwell E, Koyanagi A, Nielsen L, Hinrichsen C, Meilstrup C, Madsen KR, Koushede V (2020). Social disconnectedness, perceived isolation, and symptoms of depression and anxiety among older Americans (NSHAP): a longitudinal mediation analysis. Lancet Public Health.

[11] Sutin A, Stephan Y, Luchetti M, Terracciano A (2018). Loneliness and Risk of Dementia. J Gerontol Ser B.

[12] Simard J, Volicer L (2020). Loneliness and Isolation in Long-term Care and the COVID-19 Pandemic. J Am Med Dir Assoc.

[13] Chopik WJ (2016). The Benefits of Social Technology Use Among Older Adults Are Mediated by Reduced Loneliness. Cyberpsychol Behav Soc Netw.

[14] Neves BB, Franz RL, Munteanu C, Baecker R (2017). Adoption and feasibility of a communication app to enhance social connectedness amongst frail institutionalized oldest old: an embedded case study. Inf Commun Soc.

[15] Lee C, Coughlin JF (2014). Perspective: Older Adults' Adoption of Technology: An Integrated Approach to Identifying Determinants and Barriers. J Prod Innov Manag.

[16] Chin WW, Johnson N, Schwarz A (2008). A Fast Form Approach to Measuring Technology Acceptance and Other Constructs. MIS Q.

[17] Venkatesh V, Davis FD (2000). A Theoretical Extension of the Technology Acceptance Model: Four Longitudinal Field Studies. Manage Sci.

[18] Barbosa Neves B, Franz R, Judges R, Beermann C, Baecker R (2019). Can Digital Technology Enhance Social Connectedness Among Older Adults? A Feasibility Study. J Appl Gerontol.

